# The Effects of NR2 Subunit-Dependent NMDA Receptor Kinetics on Synaptic Transmission and CaMKII Activation

**DOI:** 10.1371/journal.pcbi.1000208

**Published:** 2008-10-31

**Authors:** David M. Santucci, Sridhar Raghavachari

**Affiliations:** Department of Neurobiology, Duke University Medical Center, Durham, North Carolina, United States of America; UFR Biomédicale de l'Université René Descartes, France

## Abstract

*N*-Methyl-d-aspartic acid (NMDA) receptors are widely expressed in the brain and are critical for many forms of synaptic plasticity. Subtypes of the NMDA receptor NR2 subunit are differentially expressed during development; in the forebrain, the NR2B receptor is dominant early in development, and later both NR2A and NR2B are expressed. In heterologous expression systems, NR2A-containing receptors open more reliably and show much faster opening and closing kinetics than do NR2B-containing receptors. However, conflicting data, showing similar open probabilities, exist for receptors expressed in neurons. Similarly, studies of synaptic plasticity have produced divergent results, with some showing that only NR2A-containing receptors can drive long-term potentiation and others showing that either subtype is capable of driving potentiation. In order to address these conflicting results as well as open questions about the number and location of functional receptors in the synapse, we constructed a Monte Carlo model of glutamate release, diffusion, and binding to NMDA receptors and of receptor opening and closing as well as a model of the activation of calcium-calmodulin kinase II, an enzyme critical for induction of synaptic plasticity, by NMDA receptor-mediated calcium influx. Our results suggest that the conflicting data concerning receptor open probabilities can be resolved, with NR2A- and NR2B-containing receptors having very different opening probabilities. They also support the conclusion that receptors containing either subtype can drive long-term potentiation. We also are able to estimate the number of functional receptors at a synapse from experimental data. Finally, in our models, the opening of NR2B-containing receptors is highly dependent on the location of the receptor relative to the site of glutamate release whereas the opening of NR2A-containing receptors is not. These results help to clarify the previous findings and suggest future experiments to address open questions concerning NMDA receptor function.

## Author Summary

Information processing in the brain is carried out by networks of neurons connected by synapses. Synapses can change strength, allowing these networks to adapt and learn, in a process known as synaptic plasticity. At a synapse, an electrical signal in one neuron is converted into a chemical signal, carried by a neurotransmitter, which is in turn converted into electrical and chemical signals in another neuron by specialized proteins called receptors. One such protein, the *N*-methyl-d-aspartic acid (NMDA) receptor, is particularly important for plasticity, due to its ability to detect the voltage of the cell receiving the neurotransmitter signal and to the fact that it allows calcium, an important signaling molecule, to enter the cell. Here we use computational modeling to investigate the role of one part of the NMDA receptor: the NR2 subunit. The subunit has various forms, and which of these forms are present in the NMDA receptor can strongly affect the kinetics and other properties of the receptor. We show that, along with changing the kinetics of the receptor, changing the NR2 subunit affects the reliability of the receptor, its ability to respond to large stimuli, and its spatial response properties. These results have implications for synaptic transmission and plasticity.

## Introduction

Excitatory synapses onto hippocampal CA1 pyramidal cells are a well-studied model system for understanding synaptic transmission and plasticity in the central nervous system [1]. These synapses contain two types of ionotropic receptors activated by the neurotransmitter glutamate: the fast α-amino-3-hydroxy-5-methyl-4-isoxazolepropionic acid (AMPA) receptor and the slower *N*-methyl-d-aspartate (NMDA) receptor [2]. The NMDA receptor (NMDAR) is permeable to Ca^2+^, which in turn drives multiple forms of synaptic plasticity [3]–[6] thought to underlie forms of learning and memory [1]. In addition to its role in plasticity, slow NMDAR currents help shape the dynamic activity of neurons and neural networks [7],[8].

The NMDAR is a multimer, composed of two obligatory NR1 subunits and two (or more) NR2 subunits [9]. The NR2 subunit exists in multiple isoforms. In the mammalian forebrain, the majority of NR2 subunits are of the NR2A or NR2B subtype [9]. The expression levels of the NR2 subtypes is developmentally regulated [10]. At birth, the NR2B subtype is dominant and there is very little NR2A expression. During the course of development, NR2A expression gradually rises to adult levels. NR2A- and NR2B-containing receptors may exhibit differences in their spatial localization [11]–[14] and may also vary in relative numbers across synapses [15]–[17]. NR2 subunit identity can also confer very different biophysical properties onto the NMDA receptor [9],[18]. Differences in subunit composition could thus have important consequences for synaptic plasticity and neuronal function. Notwithstanding the importance of these issues, fundamental questions concerning the activation properties of NMDA receptors containing different NR2 subtypes remain open.

One question concerns the fidelity with which NMDARs of distinct subunit composition at the synapse respond to glutamate release. Some studies have suggested that the open probabilities of NR2A- and NR2B-containing receptors are similar [19], while others indicate that they are dramatically different [20],[21]. Pharmacological isolation of each receptor subtype could potentially resolve this issue, but the available drugs for blocking NR2A-containing receptors are not specific enough [22]–[24]. A second question concerns the number of functional NMDARs at a synapse. Measurement of NMDAR activation at single spines using two-photon glutamate uncaging and calcium imaging has begun to address this issue [17],[25],[26]. However, it is not known how these measurements would be affected by differences in NR2 subunit composition. Other open questions concern the spatial distribution of NMDARs containing different NR2 subtypes in and out of synapses and the response of receptors at different locations to glutamate release. These may have particular bearing on experiments that suggest spontaneously released vesicles activate a different population of synaptic receptors from those activated by vesicles released due to action potentials [13],[27]. The role of NR2 subunit identity in long-term potentiation is also an open question, as the experimental evidence is contradictory [23],[28].

Computational models with parameters well-constrained by experimental measurements can shed light on these issues. Previous models examining the role of the NMDA channel in synaptic transmission [29]–[31] have not included NR2 subtype differences and therefore have been unable to address how different subtypes could contribute to the synaptic response. To better understand the potential role of differential NR2 subunit-dependent NMDAR kinetics in synaptic transmission and plasticity, we have constructed a biophysically-realistic model of a CA1 excitatory synapse, incorporating glutamate release, diffusion and binding, and NMDAR opening and closing. We then used this model to address each of the open questions mentioned above. The model allowed us to interpret and integrate previous experimental results, as well as to suggest experiments to address remaining open questions.

## Results

### NR2A-Containing NMDARs Open More Reliably and More Rapidly Than NR2B-Containing NMDARs

**Figure 1:**
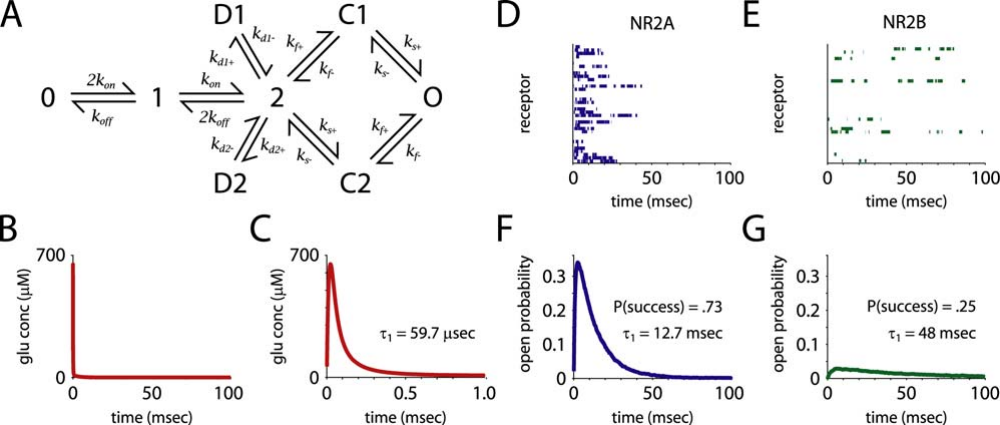
Receptor kinetics and open probability. (A) The kinetic scheme used to model the receptors. O is the open state; D1 and D2 are desensitized; 0, 1, 2, C1 and C2 are closed. Rate constants, taken from Erreger et al. [21]. and adjusted for temperature, are listed in Table 2. (B,C) Time course of free glutamate concentration in the synaptic cleft. Glutamate concentration peaked and decayed rapidly; the initial *τ* of decay was ∼60 µsec. (D,E) Examples of single receptor opening and closing in response to glutamate release show that NR2A-containing receptors fail less often, and open and close more rapidly than NR2B-containing receptors. Note that the simulations were carried out at 33°C, so the receptor kinetics are significantly faster than those observed by Erreger et al. [21]. (F,G) Peak open probability was 10 times higher, the early decay component time constant was four times faster, and the probability of success a receptor opening in response to glutamate release (P(success)) was three times greater for NR2A-containing receptors than for NR2B-containing receptors.

Previous models of NMDA receptors [30]–[33] have been based on the kinetic schemes derived from recordings by Lester and Jahr [29]. Since then, genetic techniques have allowed for a more detailed understanding of the biophysics of NMDA channel gating [21],[34],[35]. Erreger et al. [21] measured single-channel NMDAR kinetics of recombinant diheteromeric NMDARs by expressing NR1 with either NR2A or NR2B. These recordings were used to fit kinetic schemes for each receptor type (Figure 1E) and predicted the average behavior of the channels in response to brief glutamate pulses. The surprising result from this study was that the kinetics of previous models of NMDA receptors were similar to those observed for NR2B-containing receptors, with slow opening, closing and glutamate unbinding. The NR2A-containing receptors, on the other hand, showed markedly faster kinetics, with an on-rate constant for glutamate (∼3×10^7^/M·sec) similar to that of the AMPA receptor (∼2×10^7^/M·sec). These results also implied that NR2A-containing receptors were better suited to sense rapid glutamate transients in the synaptic cleft and would open with a high probability, while NR2B receptors appeared to be tuned to sense ambient levels of glutamate and would open with much lower probability. However, the study used simplified models of glutamate concentration in the cleft and therefore may not inform us about how NMDA channels open in response to synaptically released glutamate.

We have previously developed a stochastic model of AMPA receptor transmission [36] using standard Monte Carlo techniques [31],[37],[38], which accounted for channel structure, activation and desensitization [39]–[42]. By implementing the kinetic schemes fit for NMDA receptors [21] within this biophysically-realistic Monte Carlo framework, we were able to simulate individual NMDA receptor responses to realistic glutamate signals. The model tracked the diffusion of individual glutamate molecules in a structurally-constrained model of the synapse. The simulated synapse had dimensions corresponding to an average nonperforated synapse on a mushroom or stubby spine [43]. Glutamate was released from a vesicle and diffused through a fusion pore out into the cleft, where it diffused out into the extrasynaptic space, potentially interacting with the receptors on its way. Parameters used for the simulation of glutamate diffusion are listed in Table 1. The temperature-adjusted rate constants of the NMDAR model are listed in Table 2.

**Table 1:** Parameters used in simulation of glutamate diffusion.

Parameter	Value	Notes	References
Temperature	33°C		
*Q*_10_ (diffusion)	1.4/10°C		
*Q*_10_ (rate constants)	2.2/10°C		[110]
Diffusion coefficient	5.0 cm^2^/s	7.6 cm^2^/s in free solution	[106]
Cleft width	15 nm		[38]
Synapse area	0.12 µm^2^		[43]
Glutamate in vesicle	2000	200 mM	[119]
Transporter membrane fraction	0.1	10000/µm^2^	[111]
Transporter binding rate	3.2×10^7^/M·sec		[112]
Transporter unbinding rate	3016/sec		[112]
Transporter transport rate	905/sec		[112]

**Table 2:** Rate constants for NMDAR models.

Parameter	NR2A-NMDAR	NR2B-NMDAR	Triheteromer
*k*_on_	50.6×10^6^/M·sec	4.53×10^6^/M·sec	
*k*_off_	3046/sec	115/sec	
*k_f_*+	9469/sec	8553/sec	9011/sec
*k_s_*+	694/sec	145/sec	443/sec
*k_f_*−	525/sec	528/sec	526/sec
*k_s_*−	537/sec	694/sec	591/sec
*k_d_*_1_+	257/sec	1659/sec	932/sec
*k_d_*_2_+	694/sec	338/sec	516/sec
*k_d_*_1_−	89.6/sec	245/sec	194/sec
*k_d_*_2_−	3.05/sec	2.74/sec	2.94/sec

**Figure 2:**
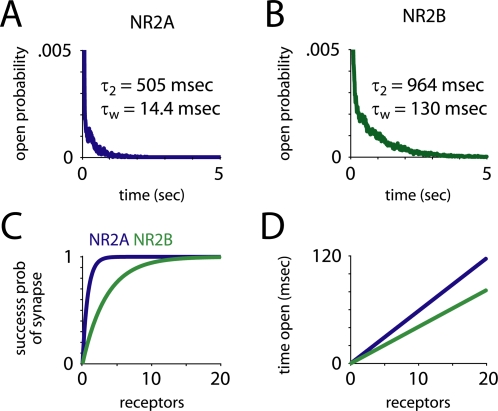
Time open and receptor failure. (A,B) Upon opening, NR2B-containing receptors stayed open much longer than NR2A-containing receptors. The late decay component time constant was twice as slow, and the weighted time constant of decay (*τ_w_*) was 10 times slower. (C) The probability of at least one receptor opening, given the number of receptors at the synapse, shows that very few NR2A-containing receptors are needed to provide near-perfect fidelity. (D) Total time open, given the number of receptors. Despite the fact that NR2A-containing receptors open three times as often, NR2B-containing receptors stay open longer, so total time open is only about 50 percent greater for NR2A-containing receptors.

NR2A-containing receptors (NR2A-NMDARs) were about three times as likely as NR2B-containing receptors (NR2B-NMDARs) to open in response to the release of a single vesicle (*P* = 0.73 vs. 0.25). NR2A-NMDARs opened and closed much more quickly, and their peak open probability was more than 10 times greater (0.34 vs. 0.03, Figure 1B–E). On the other hand, NR2B-NMDARs closed more slowly than NR2A-NMDARs (*τ_w_* = 14.4 vs. 130 msec, *τ*_1_ = 12.7 vs. 47.9 msec, *τ*_2_ = 505 vs. 964 msec, Figure 2A and 2B). The weighted time constant of decay, *τ_w_*, was calculated by taking an average of the two time constants (*τ*_1_ and *τ*_2_) derived from a double exponential fit, weighted by their coefficients in that fit. When NR2B-containing NMDARs opened, they spent twice as much time open (8.0 vs. 16 msec) as NR2A-containing receptors. Thus, the overall time open and average open probability were only about 50 percent greater for NR2A-NMDARs (time open = 5.9 vs. 4.1 msec, Figure 2D).

### The Apparent Open Probability of NR2A- and NR2B-Containing NMDARs in Neurons

Single-channel measurements of cloned channels have shown that, as in our model, NR2A-containing receptors have a greater probability of opening than NR2B-containing receptors [21],[22]. However, the results of another study suggests that this may not be the case for receptors expressed in neurons. Prybylowski et al. [19] overexpressed either the NR2A or the NR2B NMDAR subunit in cultured cerebellar granule neurons and recorded NMDAR currents from nucleated patches in response to brief applications of glutamate and MK-801, a very high affinity open-channel NMDAR blocker. The decrease in current over successive stimulations, which reflected the open probability during the previous applications, was essentially the same in the control and in both of the overexpression conditions. This is an indirect measurement of open probability however, and the lack of observed differences between conditions could be explained by a number of other factors. First, the affinities of MK-801 with channels containing different subtypes are not the same. Dravid et al. [44] reported that the IC_50_ for NR2A-containing NMDA receptors was 4.5 times greater than for NR2B-containing receptors, while fitted kinetic schemes showed similar off-rate constants for the two receptor types. Thus, the on-rate constant should be about 4.5 times faster for NR2B-NMDARs, so they will be blocked faster for the same open probability. Second, the measure of open probability chosen to quantify the response, peak current, will produce a measurement that disproportionately reflects the opening of NR2A-containing receptors, due to their much faster opening and higher peak open probability.

**Figure 3:**
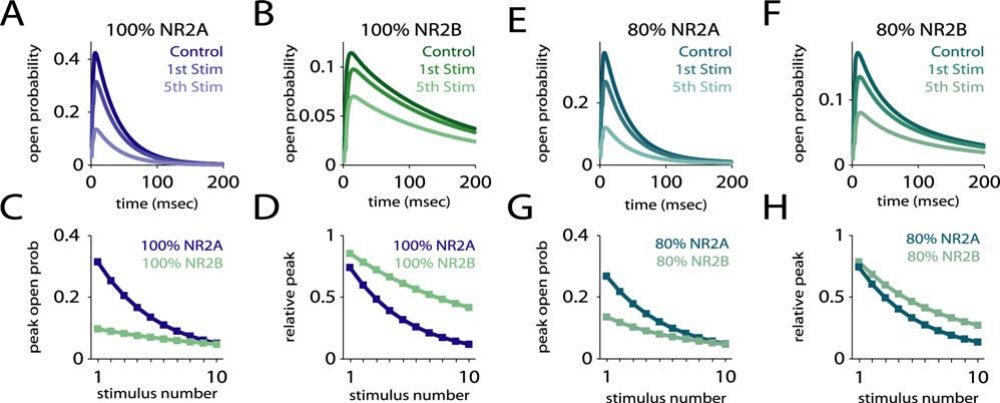
Estimating open probability using MK-801 block. Simulation of an experiment that used brief pulses of glutamate and MK-801 to estimate the open probability of receptors given different NR2A/NR2B ratios [19]. The average behavior of NR2A and NR2B-containing receptors under this protocol was simulated using a probabilistic model. (A,B) The responses of NR2A and NR2B-containing receptors alone, showing the responses to glutamate alone (Control) and to the 1st and 5th stimulations. (C,D) The peak open probability upon successive stimulations, unnormalized (C) and normalized relative to the response to glutamate alone (D), showing that NR2A-containing receptors had a higher open probability, and were blocked more rapidly. (E–H) Same as above, but for two mixed populations of receptors. A population containing 80 percent NR2A-containing receptors had a higher open probability and was blocked more rapidly than a population containing 80 percent NR2B-containing receptors. However, when plotted relative to control (H), the block appeared very similar in the two cases. Similar results were observed for other mixed populations.

To examine how these factors may have affected the results of Prybylowski et al. [19], we constructed a simulation of their experiment (Figure 3), using a modified version of our NMDAR kinetic schemes and the kinetic parameters from Dravid et al. [44] for the block of NMDARs by MK-801. The kinetic scheme was essentially a doubled version of the eight-state kinetic scheme, with a blocked and unblocked version of each of the eight states and a single, reversible connection between the blocked and unblocked open states. When MK-801 is bound to the receptor and the receptor is no longer in the open state, MK-801 becomes trapped, so both blocking and unblocking are glutamate-dependent. We used IC_50_ values (18 and 4 nM for NR2A- and NR2B-NMDARs, respectively) for resting membrane voltage [44], and ran the simulation at 23°C [19]. We applied a single, 4 msec pulse of 1 mM glutamate, followed ten pulses of 200 µM MK-801 and 1 mM glutamate [19], spaced 10 seconds apart. We set the single free parameter, the off-rate constant for MK-801 (0.25/sec), so as to produce a block after the first stimulation similar to what was observed experimentally. The simulation was deterministic, and reproduced the probabilistic time evolution of receptor state. As expected, the open probabilities were quite different, with peak open probabilities of 0.42 for NR2A-NMDARs and 0.11 for NR2B-NMDARs (Figure 3C). The average percent block from one stimulation to the next was also quite different (19 vs. 8.4 percent). When plotted relative to the peak open probability of the control stimulus, the slope of the change in peak open probability is initially higher for NR2A-containing receptors, but tapers off to a level similar to that of NR2B-containing receptors (Figure 3D). To approximate the overexpression cases of Prybylowski et al. [19], we considered the case of 80 percent NR2A-NMDARs and 20 percent NR2B-NMDARs versus 80 percent NR2B-NMDARs and 20 percent NR2A-NMDARs. The relative expression of the different subunit types in the experiment were unknown, so these cases were chosen to represent high and low expression cases; other choices yielded similar results. The normalized decline in peak open probability was very similar in the two cases (Figure 3H). As in the experimental results, the slope of the decline was initially steeper for the NR2A “overexpression” case (−0.11 vs. −0.086/stimulation for stimuli 1–4), but similar later (−0.039 vs. −0.038 for stimuli 5–10). The one feature observed by Prybylowski et al. [19] that our simulations did not reproduce was a larger relative block of NR2B-containing receptors after the first stimulation, impossible given the steeper initial decline for NR2A-containing receptors. However, Monte Carlo simulations of the experiment showed a high degree of trial-to-trial variability, so it is possible this feature was simply due to random trial-to-trial variation in the experiments (Prybylowski et al. [19] did not report the number of trials or show error bars for their data).

### Multivesicular Release and NMDAR Saturation

**Figure 4:**
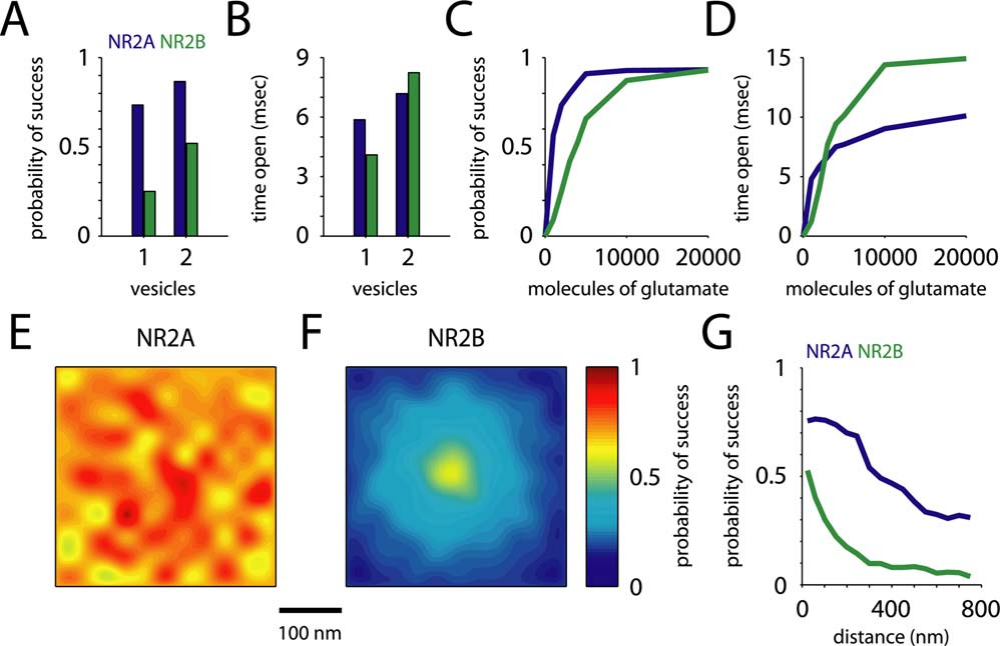
Spatial pattern of receptor opening. (A,B) Probability of opening (A) for NR2B-containing NMDARs nearly doubled, while increasing only modestly for NR2A-containing NMDARs for simultaneous release of two vesicles. Average time (B) open for NR2B-containing receptors was consequently greater. (C,D) The probability of receptor opening (C) and time open (D) versus the number of glutamate molecules released from a single vesicle increased linearly for NR2B-containing NMDARs up to 5000 molecules, at which point NR2A-containing NMDARs had already began to saturate. At 3000 molecules, the total time open for NR2B- was greater than for NR2A-containing NMDARs. (E,F) NR2A-containing NMDAR success probability (A) was nearly indifferent to location, while NR2B-NMDARs (B) showed a hot spot of success probability near the site of glutamate release. (G) The decrease in success probability with distance from the site of release for both synaptic (distance from release site<250 nm) and perisynaptic receptors.

Multiple lines of evidence have shown that multivesicular release, the release of more than one glutamate vesicle in response to a single action potential, may occur at central synapses [25],[45],[46]. However, little is known about the consequences for neural function. We have previously shown that AMPA receptors can respond in a nearly linear fashion to multivesicular release [36]. In order to extend these results to NMDA receptors, and compare the response of receptors with different subunit composition, we simulated multivesicular release by allowing glutamate to diffuse out of two vesicles. Results at this spatial and temporal spacing were representative of a variety of spacings; changing these parameters did not alter the results significantly. Figure 4A and 4B summarize the results. NR2B-NMDARs responded linearly, with the probability of success for two vesicles 2.1 times what it was for one (0.52 vs. 0.25), while NR2A-NMDARs showed only a modest increase in success probability (0.87 vs. 0.73, ratio = 1.2). Success probability was still 67 percent greater for NR2A- than for NR2B-NMDARs, but time open was 15 percent longer for NR2B-NMDARs (Figure 4B). Chavis and Westbrook [47] found a population of synapses expressing NR2B-containing NMDARs early in development that showed a high probability of NMDA receptor activation. These responses may have been due to an increased probability of multivesicular release at these synapses, or to an increase in the number of glutamate molecules per release event [48], typically denoted as the quantal size, *q*.

The reason NR2B-containing receptors are able to respond linearly to multivesicular release and NR2A-NMDARs are not is relatively intuitive. Since 75 percent of NR2A-NMDARs open in response to a single vesicle release, not many receptors are available to respond to the additional glutamate. NR2A-NMDARs, on the other hand, have a low probability of opening, so there are enough receptors available to produce a graded response. This may seem counterintuitive given the higher affinity of NR2B-NMDARs, but the conditions simulated are far from steady state, so the dynamic properties of the receptors, rather than their steady-state properties, determine their behavior. To further explore how receptor saturation shaped the NMDAR responses, we released a glutamate from a single vesicle and varied *q* from 1000 to 20000 molecules. While this is not a physiological value for *q*, it does illustrate how NR2B-NMDAR responses saturate at much higher glutamate levels. Figure 4C and 4D show how success probability and time open increase with the number of glutamate molecules released. NR2A-containing NMDAR success probability reaches 90 percent of its saturated level with only 4000 glutamate released, but NR2B-containing receptors do not reach 90 percent until 10000 glutamate, or the equivalent of five vesicles, are released. This is in agreement with experimental results showing that NMDA responses were not saturated by single release events [49] and previous simulations where only a single receptor subtype (NR2B) was considered [31]. Our results suggest that at synapses where multivesicular release can occur, or even where the glutamate content of single vesicles is variable, NR2B-containing NMDARs could be very important for the transduction of graded glutamate signals.

### Location Dependence of NMDAR Opening

We next studied the location-dependence of NMDAR activation relative to the site of glutamate release. When glutamate is released from a vesicle in the active zone, a very short-lived, high-concentration “hot spot” is produced in the synaptic cleft. AMPA receptors are very sensitive to this, and their probability of opening shows a similar hot spot around the site of release [36]. We would expect NMDARs, which have a higher affinity and lower desensitization, to open in response to more distant glutamate release, but we would also expect distinct differences between receptors containing different NR2 subtypes, due to their different kinetics. To investigate this, we compared the response of receptors in our model located close to the release site with the response of those located farther away. We held the site of release constant, close to the center of the synapse, and randomly varied the locations of the receptors. Figure 4E and 4F show the probability of receptor opening as a function of position in the cleft. NR2B-containing receptors showed an activation hot spot similar to that of AMPA receptors, while NR2A-NMDARs were almost indifferent to location. The NR2B-NMDARs closest to the release site (mean distance = 44 nm, *P* = 0.46) were more than three times as likely to open as those farthest away (mean distance = 228 nm, *P* = 0.14). For NR2A-NMDARs, the difference was less than 10 percent. The intuitive explanation of these response properties depends, again, on the dynamic properties of the receptors. While under steady-state conditions NR2B-NMDARs respond to lower concentrations of glutamate, under the conditions of a short-lived, high concentration glutamate signal the fast on-rate constant of NR2A-NMDARs that allows them to respond to lower concentrations of glutamate. Given the observed differences in location-dependence, regulation of the location of NR2B-containing receptors could have a profound effect on NMDAR transmission and synaptic plasticity. Indeed, an electrophysiological study in knockout mice showed that the location of NR2B-NMDARs may indeed be developmentally regulated [13].

NMDA receptors, primarily of the NR2B-containing subtype, can also be found extrasynaptically [50]. Recent studies have shown that the extrasynaptic pool of NMDARs activate signaling pathways that are distinct from and even opposite to the ones activated by the synaptic pool [51]. We simulated the effect of synaptic glutamate release on NMDA receptors located outside of the synaptic cleft but adjacent to the synapse, at distances of 300–750 nm from the release site. The results are summarized in Figure 4G. The probability of success of extrasynaptic NR2A-NMDARs fell off rapidly at the edge of the synapse, but was still 0.3 at 750 nm. Extrasynaptic NR2A-containing receptors have been reported recently [14], but they are probably quite rare, and a function has not been proposed for them. Our results suggest that if they are located in the vicinity of synapses, they should be fairly sensitive to single release events. NR2B-containing receptors, on the other hand, already had a low probability of opening at the edge of the synapse, which dropped to 0.04 at 750 nm. Individual extrasynaptic NR2B-NMDARs would be unlikely to open in response to glutamate release, and significant activation of extrasynaptic receptors would require that glutamate diffuse over an area of membrane large enough to contain a number of receptors. A number of studies have shown evidence of extrasynaptic NMDAR activation [33],[52],[53], suggesting that this may be the case. Our estimates of NR2B activation by efflux of glutamate from the cleft after the release of a single vesicle are similar to previous simulations [31],[54] but somewhat lower than other models where glutamate diffusion occurs in a neuropil modeled as a porous medium [33],[55]. This difference is due to assumptions about the amount of glutamate released by a single vesicle.

### The Number of NMDARs at Hippocampal Synapses

The number of NMDARs at individual synapses has been estimated from electron microscopy studies [56]. More recently, a tissue preparation technique which provides near one-to-one labeling of receptors present has provided approximate lower and upper bounds (10 and 100) for this number [57]. However, these anatomical techniques cannot distinguish functional receptors [58]. Physiological measurements [2] can in principle yield the number of active NMDAR by comparing miniature EPSCs to single channel currents. However, dendritic spines are far too small to record from individually, and techniques such as minimal stimulation do not reliably isolate single synapses [59], so an alternative approach must be used. Two-photon glutamate uncaging [17],[60],[61] or calcium imaging [17],[26] can be used to record synaptic activity at single dendritic spines.

Nimchinsky et al. [26] used calcium imaging to estimate the number of NMDARs at hippocampal synapses. They measured the frequency of synaptic failures in the presence and in the absence of D-CPP, a competitive NMDA antagonist, and calculated *M*, the number of NMDARs present at the synapse (see Methods). However, they did not differentiate between receptors containing different NR2 subtypes. We ran our simulations at 30°C [26] and determined the probabilities of opening for NR2A-NMDARs (0.70) and NR2B-NMDARs (0.20). Using the values of vesicle release probability, mean number of receptors opening and failure rate from Nimchinsky et al. [26], and measurements of NR2 subtype-dependent D-CPP block from Lozovaya et al. [62], we estimated *M*_NR2A_ and *M*_NR2B_, the average number of receptors per synapse containing NR2A and NR2B subtypes (see Methods). We arrived at estimates of 0.63 NR2A- and 11 NR2B-NMDARs, on average, per synapse.

Because these estimates depend on opening probability, and because the probability of opening of NR2B-containing NMDARs varies so dramatically with location, assumptions about the distribution of receptors will have a strong impact on the results. We calculated the number of receptors as above, but under the assumption that NR2B-NMDARs were located either near the release site, or at the periphery of the synapse. At 30°C the success probability for NR2B-containing receptors close to the release site was 0.40, and our calculations yielded an average of 0.44 NR2A- and 4.9 NR2B-NMDARs. If NR2B-containing receptors were located at the periphery, as proposed by Tovar and Westbrook [50], probability of opening dropped to 0.11 and the number of receptors rose to 0.69 NR2A- and 19.2 NR2B-NMDARs. Our estimates compare well with the limits placed by structural [56] and two-photon imaging measurements [26]. Moreover, given the differences in *P*_open_ for the two subtypes, our models predict that blocking NR2B receptors would result in a mean reduction of 50 percent in peak current, which is consistent with experimental data [17].

### Simulations of Triheteromeric NMDARs Using a Kinetic Model

**Figure 5:**
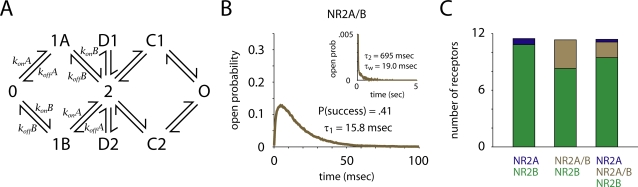
Triheteromeric receptors and the number of receptors at a synapse. (A) The kinetic scheme used to model the triheteromeric receptors. Glutamate binding and unbinding to the NR2A and NR2B subunits was independent, thus there were two single-bound states (1A, 1B). Binding and unbinding rates for NR2A and NR2B were as in the previous models; all other rate constants were the geometric means of the values in the previous models (Table 2). (B) Opening and closing kinetics for our model triheteromeric NR2A/B-containing receptor. Open probability and decay kinetics were intermediate between the diheteromeric receptors, but more similar to those of NR2A-containing receptors. (C) Average number of each type of receptor present at the synapse, based on our simulations and the results of Nimchinsky et al. [26], under three different assumptions: that only diheteromeric NMDARs were present (AA, BB), that only NR2B- and NR2A/B-containing receptors were present (AB, BB) and that all three receptor types were present and combined randomly (AA, AB, BB). In all three cases, NR2B subunits made up greater than 85 percent of the subunit content at the synapse. There was no positive solution for the case where only NR2A and NR2A/B-containing receptors were present.

Our kinetic models so far have been restricted to channels that exclusively contain NR2A or NR2B subunits. However, multimeric channels that contain both subunits are known to be present at hippocampal synapses [12],[63], although a recent study indicated that the majority of receptors are diheteromeric [64]. Up to this time, no kinetic model exists for a triheteromeric channel, as isolation of these channels for recording would be extremely difficult, though it may be possible by exploiting differential sensitivity to antagonists such as ifenprodil and zinc [12],[65],[66]. Without a kinetic scheme, it is difficult to estimate the number of these channels at synapses. As a first-pass approximation, we constructed a kinetic scheme derived from the schemes of the diheteromeric channels (Figure 5A). We assumed that glutamate bound and unbound from each subunit independently, so there were two single-bound states (1A and 1B). The rate constants for these steps were the same as the rates for NR2A and NR2B in the diheteromer models. For the other kinetic transitions, which are proposed to be due to conformational changes in the NR1 subunits, we set the forward and reverse rate constants of each state transition such that the ratio between these rates and the sum of the magnitudes of the rates were the mean of those in the NR2A- and NR2B-NMDAR models.

We used this kinetic scheme in our model to estimate the kinetics and probability of opening of these simulated triheteromeric receptors (Figure 5B). The triheteromer kinetics were intermediate between those of NR2A- and NR2B-NMDARs, but closer to those of NR2A-NMDARs. The weighted time constant of decay was 19.0 msec (*τ*_1_ = 15.8 msec, *τ*_2_ = 695 msec), the probability of success was 0.41, and the time open given a success was 7.8 msec, yielding an overall time open of 3.1 msec. If triheteromeric receptors behave similarly to those in our model, then in mature animals, where NR2A expression levels are high, a significant fraction of the NMDA receptors would have kinetics significantly faster than those assumed by most previous models, even if the majority of NR2A subunits were incorporated in triheteromeric receptors. This would have significant consequences for the results of many previous models.

Using the results from this simulation, we repeated the calculation of the mean number of receptors per synapse from the data of Nimchinsky et al. [26] (Figure 5C). We assumed that inhibition constant for D-CPP for the triheteromeric channel was the geometric mean of those for the diheteromers. To solve for the number of receptors of three species (*M*_NR2A_, *M*_NR2B_, *M*_NR2AB_), we needed an additional constraint. We calculated solutions under four different assumptions. The first, that only NR2A and NR2B receptors were present, is what we calculated earlier (*M*_NR2A_ = 0.63, *M*_NR2B_ = 11). The second, that only triheteromeric and NR2B receptors were present, yielded *M*_NR2AB_ = 3.0 and *M*_NR2B_ = 8.3. Under the assumption that only NR2A and NR2A/B receptors were present there was no positive solution. Finally, we assumed that all three types were present and that they combined randomly (that is, 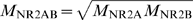). Under these conditions, *M*_NR2A_ = 0.29, *M*_NR2AB_ = 1.7 and *M*_NR2B_ = 9.4. Overall, these results indicate that NR2A subunits made up somewhere between 5 and 15 percent of the total NMDA subunits present in functional receptors. This is not particularly surprising given the developmental age of the animals, but a similar approach could be used to estimate the number of receptors present at different developmental time points. Such estimates would be valuable in understanding the role of NMDARs in adult synapses, and in understanding the role of the developmental NR2 subunit switch.

### Effects of NR2 Subtype on CaMKII Activation

Synaptic NMDARs are incorporated into multiprotein complexes and are in close proximity to many calcium-sensitive enzymes, such as Ca^2+^-Calmodulin-dependent Kinase-II (CaMKII) [67] and protein phosphatases [68]. These enzymes are ideally positioned to detect the time-varying calcium concentration changes due to influx through NMDARs and transduce these signals by altering the phosphorylation state of their various substrates. The ensuing signaling events can lead to rapid alteration in the number of AMPARs in the synapse [61], [69]–[71], activation of protein synthesis machinery in dendrites [72] and gene transcription in nucleus important for long-term maintenance of neuronal plasticity [73]. A number of recent studies have suggested that NR2A- and NR2B-containing NMDA receptors selectively induce potentiation and depression, respectively, of hippocampal synapses [28],[74],[75]. However, other studies have suggested that either subtype can be sufficient for the induction of long-term potentiation [23],[76], that NR2B-containing receptors can drive LTP [77],[78], or that either subtype can drive long-term depression [79]. In many of these studies, differences were seen depending on developmental age and induction protocol. The differences in the ability of the receptor subtypes to induce plasticity could arise due to their distinct kinetics, which result in distinct spatiotemporal pattern of calcium concentration in the postsynapse. The rapid, reliable opening of NR2A-containing NMDA receptors, would produce large rapid increases in internal Ca^2+^ concentrations, which has been shown to selectively lead to LTP [80]. On the other hand, the much longer-lived activation of NR2B-containing NMDARs could lead to enhanced potentiation in situations where depolarization occurs over a long period of time, such as during bursts. On average, NR2B-containing NMDA receptors let in as much or more Ca^2+^ than NR2A-containing NMDARs, but they also fail much more often. Therefore, the variability in the Ca^2+^ signal through NR2B-containing NMDARs is very high. This variability could have significant effects on LTP induction. We next explored these questions by coupling our model of NMDAR activation to a postsynaptic model of LTP.

We calculated calcium influx from receptor opening data from our Monte Carlo simulation and used it as the input to a model of a CaMKII switch [81]. This latter model is deterministic, and assumed that all reactions are taking place in a single, well-stirred compartment. Each molecular species was represented by a single, time-varying concentration, and the model was a system of differential equations relating those concentrations. In the model, CaMKII activation was bistable between an unphosphorylated state and an activated state where a large fraction of CaMKII subunits are phosphorylated (see Methods). Its activation is set by the balance of the rates of calcium-dependent phosphorylation and autophosphorylation with the rate of dephosphorylation by Protein Phosphatase 1 (PP1). Under baseline conditions, dephosphorylation is faster than phosphorylation and activity tends towards a low level. Once calcium-dependent phosphorylation pushes the level of activation above a threshold, autophosphorylation begins to out-compete dephosphorylation, and CaMKII activity tends towards a high level.

**Figure 6:**
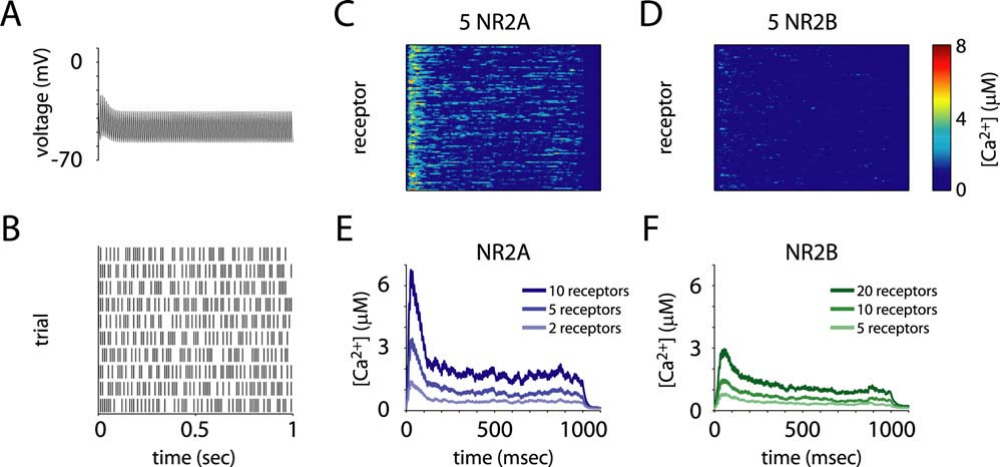
Calcium influx in response to tetanic stimulation. (A,B) LTP was induced by 100 Hz tetanic stimulation with a duration of 1 sec. The postsynaptic voltage (A) and stochastic glutamate release (B) were both modulated synaptic facilitation and depression. (C–F) Example postsynaptic calcium concentration traces (C,D) from simulations with 5 NR2A-containing or 5 NR2B-containing NMDARs, and mean calcium concentration in the spine in for three different numbers of receptors (E,F) show that NR2A-NMDARs drove spine calcium concentration much higher, per receptor, than did NR2B-NMDARs.

Calcium current was determined by a simple model, based on the Goldman-Hodgkin-Katz equations [26]. Conductance and calcium permeabilities were the same in NR2A- and NR2B-NMDARs, as has been measured experimentally [82],[83]. We assumed the block of NMDARs by Mg^2+^ was an instantaneous, voltage-dependent process, and modeled by fitting a sigmoidal curve to fractional block versus voltage data [83]. Our LTP induction protocol (Figure 6A and 6B) consisted of a train of 100 stimuli delivered at 100 Hz. The synapse had 60 percent release failure and both facilitation and depression were modeled [84], based on measured values [85],[86]. We modeled postsynaptic voltage using a simple, single exponential approximation of the results of a detailed simulation [87]. For each stimulus, the voltage exponentially approached −10 mV for 1 msec with a time constant of 0.1 msec and fell back towards the resting voltage with a time constant of 9 msec.

**Figure 7:**
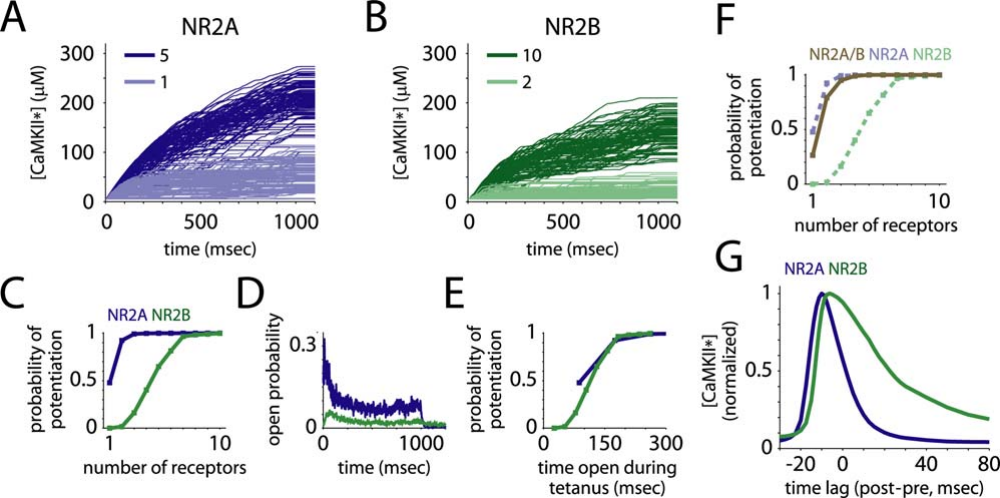
Effects of NR2 subtype on long-term potentiation. The synapse model was coupled to a model of postsynaptic potentiation, where CaMKII phosphorylation is the switch for LTP. (A–C) Sample traces of active CaMKII concentration for synapses with different numbers of each receptor type present (A,B) show that NR2A-containing receptors drove LTP more effectively, per receptor, than did NR2B-containing NMDARs (C). (D) The receptor open probability during the stimulation period was the main determinant of calcium influx. (E) The total time the receptors spent open during the stimulation was a good predictor of the probability a synapse would be potentiated. (F) Long-term potentiation via the triheteromeric receptors was also intermediate between NR2A- and NR2B-containing receptors but more similar to NR2A-containing NMDARs. (G) When glutamate release was paired with brief depolarizations of the postsynaptic cell at different temporal offsets, NR2A-containing receptors showed a much narrower temporal window for potentiation than did NR2B-containing receptors.

NR2A-containing NMDARs let in more calcium per receptor than NR2B-NMDARs (Figure 6C–F), and were more effective at driving LTP (Figure 7A–E). The probability of a synapse to potentiate after tetanic stimulation exceeded 99 percent with only 3 NR2A-NMDARs present, while the same required 9 NR2B-NMDARs (Figure 7C). Even if we set the number of receptors such that the total time open was the same, NR2A-NMDARs showed a greater rate of potentiation. This is because the time they spent open was mostly right after glutamate release, while the postsynaptic cell was depolarized. The total time open during the one second tetanic stimulation period, however, predicted the probability of potentiation well (Figure 7D and 7E). This quantity was about three times longer per receptor for NR2A-NMDARs than for NR2B-NMDARs (87.4 vs. 26.1 msec). We ran the simulation using our hypothetical kinetic scheme for triheteromeric receptors. Again, the behavior of the NR2A/B receptors was intermediate between the diheteromers but more similar to that of NR2A-NMDARs (Figure 7F). Reaching a 99 percent probability of potentiation required 4 receptors, and the time open during the tetanus also predicted the probability of potentiation well.

The precise timing of postsynaptic spikes relative to presynaptic glutamate release can have drastic effects on the magnitude and direction of synaptic potentiation [88]. Because the opening of NR2A- and NR2B-containing NMDARs have very different time courses, they may show great differences in this kind of precise timing-dependent plasticity. To test this, we paired 50 presynaptic glutamate release events with 50 postsynaptic voltage spikes, and varied the relative timing between them (Figure 7G). NR2B-NMDARs showed a much broader window in which paired stimuli could still drive LTP, while NR2A-NMDARs required relatively precise timing. The width at half height for NR2B-NMDARs was twice that of NR2A-NMDARs (∼36 vs. 18 msec). This suggests that the NR2 subunit may play an important role in determining the spike timing-dependent properties of LTP.

## Discussion

In this study, we considered the role of NMDA receptor NR2 subunit on synaptic transmission and synaptic plasticity. We used a kinetic model of NMDA receptors [21] in a model of a central nervous system excitatory synapse whose parameters were well constrained by experimental measurements. We explicitly modeled the release of the neurotransmitter glutamate from a synaptic vesicle, its diffusion in and out of the cleft, its binding to NMDA receptors, and the opening and closing of the receptors. We showed that NR2 subunit composition dramatically affects the probability and the spatiotemporal pattern of synaptic receptor activation which can have significant effects on the activation of signal transduction events downstream of receptor activation.

### Fidelity and Kinetics of Receptor Opening

We found that NR2A-containing receptors were about three times as likely as NR2B-containing receptors to open in response to a single glutamate vesicle release. This is in agreement with previous, *in vitro* studies [20],[21]. In addition, when NR2B receptors opened, their total time open was about twice as long on average as NR2A-containing receptors, so the trial-to-trial variability in time open was much greater. The kinetics of the NR2B-containing receptors were much slower, however, and receptor opening was spread out over a much longer time. The peak open probability was more than 10 times greater for NR2A-containing receptors than for NR2B-containing receptors, while the weighted time constant of decay was almost 10 times slower. This distinction between different measures of open probability is important for the interpretation of experimental results. For example, a number of studies have used progressive blockade of NMDAR excitatory post-synaptic currents (EPSCs) by MK-801 to estimate open probability [19],[33],[47]. MK-801 is an irreversible open channel blocker, so progressive blockade reflects prior NMDAR opening. Typically, it is interpreted to indicate success probability, or even the number of receptors activated. However, blockade is usually measured relative to a baseline, and blockade is not instantaneous, so in the case of a chronic application of MK-801, blockade is actually indicative of mean time open. Thus, our results agree with Scimemi et al. [33], who showed that MK-801 blocked NR2B-containing NMDARs faster than NR2A-containing NMDARs. In a study where MK-801 is applied briefly [19], blockade should better indicate peak open probability, although it may not if washout is incomplete.

Our results were based on receptor kinetics measured in a heterologous system. However, it has proved much more difficult to determine the properties of NMDA receptor subtypes natively expressed by neurons. One study that attempted to do so [19] implied that there was no difference in average open probability between NR2A- and NR2B-containing receptors. However, other studies suggest that the differences in activation kinetics between the two receptor subtypes measured *in situ* were similar to those measured *in vitro*. Our simulations of the Prybylowski et al. [19] experiment predicts that this result could potentially have arisen despite a difference in open probability, due to differences in antagonist affinity and the way receptor block was quantified. The presence of triheteromeric receptors could further complicate this situation. To finally resolve the question of the open probabilities and kinetics of NMDA receptors in synapses, direct measurements will have to be made. However, this has proved elusive, primarily due to the lack of a selective blocker for NR2A-containing receptors. However, genetic methods can be used to isolate receptor subpopulations [66],[89]. In combination with two-photon uncaging and/or imaging [17],[26], these methods should allow the properties of NMDA receptors to be measured *in situ* and the predictions of our model to be tested.

The slower opening kinetics of NR2B-containing receptors could have important consequences for calcium influx during miniature excitatory postsynaptic events. Due to the small size of the dendritic spine and the high resistance of the spine neck [90], AMPA mEPSCs should be sufficient to depolarize the spine head and relieve the NMDAR Mg^2+^ block. As the peak open probability of the NR2A-containing receptor is much greater than that of the NR2B-containing receptor, the Ca^2+^ influx during the brief AMPA mEPSC would be much greater. It has been shown that NMDA miniature excitatory currents can stabilize synaptic strength [72]. One prediction of our model is that the homeostatic stabilization of AMPA receptors at the synapse is preferentially mediated by NR2A-containing NMDARs. This prediction could be tested by studying whether homeostatic stabilization in the presence of ifenprodil (which blocks NR2B containing receptors) differs from that in NR2A knockout animals.

### Location and Number of Receptors

We studied the differences in receptor activation as a function of the amount of neurotransmitter released. Such differences can arise either due to variation in the amount of neurotransmitter contained in vesicles [31],[91] or differences in the number of vesicles released [25],[45],[46]. We found that synapses with predominantly NR2A-containing receptors were nearly insensitive to differences in the amount of neurotransmitter, while those with predominantly NR2B-containing receptors responded in graded fashion (Figure 3). We next considered the impact of distance from the release site on the activation of NMDA receptors containing different subtypes. While NR2A-containing receptors responded about equally regardless of where they were in the synapse, NR2B-containing receptors were highly location-sensitive, with the receptors located closest to the release site opening three times as often as the receptors located farthest away. Receptors located perisynaptically, outside the synapse but less than 1 µm from the release site, showed a very low probability of opening.

These results have important implications for the hypothesis that, over the course of development, NR2B-containing receptors at the center of synapses are displaced by NR2A-containing receptors, such that NR2B-containing receptors end up preferentially located at the periphery of synapses. This idea is based on the finding that miniature excitatory post-synaptic currents (mEPSCs) progressively declined with age in NR2A knockout animals, while evoked activity, which could result in multivesicular release, could still produce an NMDA current [13]. Our results suggest that NR2B-containing receptors located at the periphery of synapses would be very unlikely to open, even under evoked activity. This is difficult to reconcile with the experiments of Townsend et al. [13]. One potential explanation is that the mEPSCs of single NR2B-containing receptors are difficult to distinguish from noise, due to the extended, rapidly opening and closing nature of their activation. In the knockout experiments, the number of NR2B-NMDARs decreased over development. If the number of NR2B-NMDARs per synapse is relatively low, spontaneous release would be likely to open only one, or zero, NR2B-containing receptors, making mEPSCs nearly impossible to detect. On the other hand, action potential evoked release, might be multivesicular, leading to the activation of a detectable number of receptors (Figure 4). Once again, further experiments are needed to test the hypothesis.

Our simulations suggest one such experimental test. NR2B-NMDAR exhibit a sharp location-dependence of opening probability, implying that they require a very high concentration of glutamate to open. NR2A-NMDARs are essentially location-independent, suggesting their response is essentially saturated at low concentrations. Thus, a low-affinity antagonist such as L-AP5 or D-AA would have a much more dramatic effect on NR2B-containing receptors. Similarly, the antagonist would be much more effective at blocking NR2B-NMDARs located at the periphery of the synapse. If, later in development, NR2B-containing receptors are not just decreasing in number but are preferentially located at the periphery, we would expect a proportionally much stronger block of evoked activity when applying a low-affinity antagonist. A related prediction of our model is that in addition to the progressive decline in the spontaneous NMDA current in the knockout animal, the variance of the evoked NMDA response should increase.

It has also been shown that glutamate spillover from adjacent synapses can activate NMDA receptors [33],[52],[92]. Whether or not this happens depends upon the activity of glutamate transporters, the rate of glutamate diffusion, temperature, the geometry of the extracellular space and the extent of sheathing of synapses by glia. We did not address those factors here, but they have been considered elsewhere [31],[93]. However, the very low opening probability of perisynaptic NR2B-containing receptors in our simulations suggests that the excitation of these NMDA receptors by spillover from a single vesicle is quite difficult. Thus, if significant activation by spillover does occur, glutamate must diffuse far enough to potentially interact with a large number of receptors. We note that our model of the NR2B-containing receptors are similar to those used in Scimemi et al. [33], and our results on activation probabilities of these receptors are similar. However, it remains to be shown whether under normal *in vivo* conditions glutamate release at a single synapse (the conditions we simulate) can cause significant activation of extrasynaptic NMDARs. It could be that these extrasynaptic NR2B-containing NMDARs instead detect changes in ambient glutamate concentration related to average synaptic activity over longer timescales or to events that cause large amounts of glutamate to be released. Another possible function for these receptors could be to detect signals originating extrasynaptically, such as glutamate release by astrocytes, which may play a role in synchronizing hippocampal pyramidal cell activity [94].

### The Effect of Subunit Composition on CaMKII Activation

We studied the potential impact of NMDAR subunit composition on postsynaptic long-term potentiation by coupling our model of receptor opening driven by tetanic stimulation to a model of activation of calcium-sensitive enzymes in the postsynapse known to be critical for induction of LTP. We found that either NR2A- or NR2B-containing receptors could drive persistent CaMKII autophosphorylation, but more NR2B receptors were required to reliably drive autophosphorylation. Similarly, the majority of experimental studies in adult animals using concentrations of NMDA receptor blockers small enough to be selective for NR2 subtype have shown that either receptor type can drive LTP [23],[95],[96], though some reports contradict this [28]. That either receptor type could drive LTP stands to reason, as the conductance and calcium permeability of both types would allow large Ca^2+^ currents to enter the postsynaptic cell while it was depolarized. We note that while our simulations suggest that given nearly equal Ca^2+^ permeabilities of the two receptor-subtypes [10],[97], NR2A-containing receptors let in more calcium than NR2B-containing NMDARs. This is compatible with experimental findings [17] which suggest that synaptic NR2B-containing receptors can have greater or smaller fractional calcium current due to post-translational modifications depending on synapse size and history [58].

In our simulation, the most important variable for determining the probability of LTP was the total time open during the period of tetanic stimulation, which was more than three times greater for NR2A-containing receptors. We would expect the advantage of NR2A-containing receptors in driving LTP to diminish in a low frequency pairing protocol where the postsynaptic cell is held at a depolarized voltage for the entire period of receptor opening, during which NR2A-containing receptors are only open for about 50 percent longer. Still, the advantage of NR2A-containing NMDARs in driving LTP is surprising, considering that NR2B-NMDARs are the dominant receptor type during early, critical periods of development [10]. It could be that early in development other forms of synaptic plasticity are dominant, or that multivesicular release is more common, there is a posttranslational modification that allows more calcium to enter [17],[58], or there is a difference in postsynaptic biochemical signaling [98]. Barria and Malinow [78] showed that in slices taken from young animals, LTP was dependent upon interaction between NR2B subunit intracellular C tails and CaMKII. This interaction may be important in allowing NR2B-containing receptors to drive LTP despite their slow kinetics. It is also interesting to note that Harris and Teyler [99] were first able to observe hippocampal LTP at P7, and that LTP was maximal at P15, time points which correspond well with the expression of NR2A.

We also found a distinct difference between the receptor types in a protocol in which glutamate release was paired with depolarization of the postsynaptic cell at different temporal offsets, similar to experiments used to assess spike timing-dependent plasticity [88]. When presynaptic glutamate release was nearly concurrent with or preceded postsynaptic depolarization by a small offset, calcium entered the postsynapse and CaMKII was autophosphorylated. But, the range of temporal offsets over which CaMKII phosphorylation occurred was about twice as wide for NR2B- as for NR2A-containing receptors, suggesting that the temporal properties of spike timing-dependent plasticity (STDP) could vary greatly with subunit composition. STDP is a competitive plasticity mechanism and could play a role in the formation and refinement of neuronal networks [100],[101].

The more permissive temporal filter of NR2B-containing receptors could allow potentially informative connections to be strengthened and stabilized initially. Later, as the network settles into a more mature state, the more precise temporal filtering of NR2A-containing receptors would allow the circuit to be refined, strengthening only the fastest and most informative of the initially stabilized connections. Another interesting temporal property of NR2B-containing NMDARs was recently reported [102]. Repeated stimulation of the receptors caused a downregulation in Ca^2+^ permeability. In combination with a permissive temporal filter, this property could allow inputs that are loosely correlated to be stabilized, while guarding against spurious connections by requiring that correlations persist over a long period of time. The same process of early, temporally-permissive filtering and later refinement could also be acting in the adult brain, where smaller, more plastic spines have longer EPSC decay times, consistent with higher NR2B content [17].

The subunit shift seen in development [10] could act as a form of temporal metaplasticity. Such a change has also been shown to take place in the visual cortex during development [103]. A recent study showed that NMDA receptor turnover was rapid and that shifts in NR2 subunit composition could occur within seconds to minutes after LTP induction [104]. Thus, the subunit shift could serve to stabilize the synaptic potentiation and as a bridge between short-term and long-term potentiation.

The NMDA receptor is, without question, one of the most important determinants of synaptic plasticity in neuronal systems, and its NR2 subunit can drastically alter its biophysical properties and determine its binding to other components of the postsynaptic density. It is surprising then, that basic pieces of information, such as the relative open probabilities of NR2A- and NR2B-containing receptors at the synapse are still unknown. Our work has attempted to address some of the ambiguities in the in the experimental data, and suggests that the open probabilities of the two receptor might indeed be quite different. In addition, it shows that the receptors are likely to vary greatly in their spatiotemporal response to glutamate release. However, these conclusions still await characterization the basic response properties of the receptors *in vivo*. Once these basic properties are characterized, many more questions will be able to be addressed, by both experimental and theoretical methods. Understanding, for example, the localization of NMDA receptors, or the role of interactions with signaling molecules in the postsynaptic density, will provide us with valuable insight into the development of neural circuits and neuronal plasticity.

## Methods

### Glutamate Diffusion

Glutamate was modeled as discrete particles, each occupying a position in 3-dimensional space not restricted to a grid. At each time step each particle took a random step, drawn from a 3-dimensional Gaussian distribution. For each dimension, the standard deviation, *σ*, was 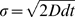, where *D* is the diffusion coefficient and *dt* is the time step. Particles moved to the endpoint of the step unless they collided with a boundary. Boundaries represented cell membranes and collisions at the boundaries were elastic, unless the particle bound to a glutamate receptor or transporter.

The space containing the synapse was modeled as two adjacent 500 nm cubes, representing the presynaptic and postsynaptic cells, contained within a larger rectangular prism. The distance across the synaptic cleft was 15 nm, as was the width of the space around the cubes. The active zone was a 350 nm square patch in the center of the cleft face of the postsynaptic cube. 121 possible receptor locations were arranged in a 35 nm-spaced grid across the active zone. On each simulation, receptors were placed randomly at these sites. The release site of the vesicle was always the same, located 18 nm from the center of the presynaptic face of the cleft. The vesicle was a 25 nm cube connected to the cleft by a fusion pore 8 nm wide and 15 nm long. Before release, the glutamate molecules were randomly placed within the vesicle. At release, they were simply allowed to begin diffusing.

The diffusion coefficient of glutamate in the neuropil has been recently estimated to be ∼3×10^−6^ cm^2^/s at the mossy fiber-granule cell synapse [105], which is 3× lower than the measured value in free solution [106]. This value was estimated by measuring the reduction of the slow AMPA-mediated EPSC, presumably activated by glutamate spillover from neighboring synapses when slices are loaded with high molecular weight dextran, a crowding agent. This reduction was then fit to a battery of glutamate receptor kinetic models to extract the best fit value of *D* that matched the observed reduction. We note that this number is an estimate, that depends on the particular kinetic model used, the amount of glutamate released per vesicle and the geometry. Direct measurements of glutamate diffusion have not been made at hippocampal synapses. Therefore, we used a similar procedure to estimate the diffusion constant of glutamate. As a constraint, we used the waveforms of sucrose-evoked AMPA miniature EPSCs measured close to the synapse [107]. We then simulated a battery of kinetic models of AMPAR activation at hippocampal synapses [36],[42],[108] in response to synaptic release of glutamate and matched the amplitude, rise-time and decay times of the mEPSC.

The AMPAR model of Jonas et al. [108] assumed that the binding of two glutamate molecules to the receptor was sufficient to activate the receptor and postulated that the binding was cooperative. This has been ruled out by subsequent experiments [39],[40], but we included it since it is the only published model of hippocampal AMPARs. The AMPAR model of Raghavachari and Lisman [36] was based on validated fits of fast glutamate application to AMPARs to outside out patches pulled from CA1 pyramidal neurons [109]. The model of [42], although originally formulated for cerebellar AMPARs, was included for completeness as it is the only kinetic scheme that accounts for multiple glutamate binding and conductance states of the receptor.

The free variables were the diffusion constant of glutamate and the number of glutamate molecules in a vesicle. The best fit values for these parameters resulted in a diffusion coefficient of 5.0×10^−6^ cm^2^/sec at 37°C. We note that these values lie at the upper end of the estimates of Nielsen et al. [105]. Moreover, the higher values of *D* in that study correlated with independent subunit models of AMPAR activation. Since our model of AMPAR activation is also an independent subunit model with multiple sub-conductance states [36], our estimate of *D* is slightly higher than that reported. Varying this value by 20 percent did not affect the simulation results qualitatively. We used a fixed time step of 0.01 µsec.

Receptors were represented by discrete 10 nm square patches on the postsynaptic membrane. When a particle hit one of these patches, a random number was generated to determine whether or not it would bind to the receptor. The probability of binding for a collision was determined by dividing *k*_on_, the number of binding events per second per M of ligand, by the expected number of collisions per second, given a ligand concentration of 1 M. The expected number of collisions per time step *dt* is half the number of particles in the volume defined by the area of the receptor and the mean step size for a particle in one dimension. So, the probability of binding was equal to 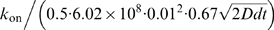.

### NMDAR Activation and Glutamate Transporters

The kinetic scheme for the NMDA receptors was as in Erreger et al. [21] (Figure 1A). There were eight states: zero bound (0), one bound (1), two bound (2), two desensitized states (D1, D2), two intermediate closed states (C1, C2) and one open state (O). The rate constants were taken from Erreger et al. [21] and adjusted for temperature (Table 2). We scaled the rate constants using *Q*_10_ = 1.4/10°C for diffusion-limited processes and *Q*_10_ = 3/10°C for non-diffusion-limited processes [110]. Our simulations were run at 33°C, so our kinetics were significantly faster than those observed by Erreger et al. [21], whose experiments were conducted at room temperature. A simulation where we fixed the temperature to 23°C, and applied a glutamate pulse of 1–4 mM for 1 msec, exactly reproduced the results of Erreger et al. [21], as it was, in fact, the same model.

Extrasynaptic membranes contained glutamate transporters. They did not have a fixed location on the membrane. Instead, we assumed the density of transporters available to bind glutamate was 10000/µm^2^ [111] and assumed that the fraction of extrasynaptic membrane was 0.1 so that when a particle hit the extrasynaptic membrane it collided with a transporter with probability 0.1. Upon collision, the probability of binding was calculated as above. On subsequent time steps a bound particle could either unbind or be transported and removed from the simulation. The rate constants of the binding, unbinding and transport steps were as in Grewer et al. [112], adjusted for temperature (Table 1).

The opening and closing of individual NMDARs was independent of the other receptors in the synapse, as assessed by varying the number of NMDARs included. The average success probability and time open of the receptors was the same whether there was one receptor present or 20. We normally included 20 receptors, and combined the receptors from each simulation into a single pool for analysis.

### Calculating the Number of Receptors at a Synapse

We calculated the average number of receptors of each per synapse based on the data of Nimchinsky et al. [26], using their equations
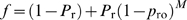
(1)

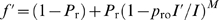
(2)where *f* is failure probability with no antagonist, *f*′ is failure probability in the presence of the antagonist, *P*_r_ is the probability of neurotransmitter release, *p*_ro_ is the probability a receptor will open given neurotransmitter release, *I*′/*I* is the ratio of the NMDA current amplitude in the presence of the antagonist to the amplitude in the absence of the antagonist, and *M* is the total number of receptors present at the synapse. They could not measure *P*_r_ directly, so they used the approximation 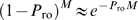 and solved
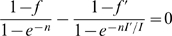
(3)numerically, where *n* = *MP*_ro_.

Nimchinsky et al. [26] report that *n* = 3.1, and by inspecting their data, we can observe that their mean *f* = 0.43, *f*′ = 0.58, and *I*′/*I* = 0.41, and that the concentration of D-CPP that will produce an *I*′/*I* of 0.41 is 240 nM. We can then calculate that *P*_r_ = 0.60 by *P*_r_ = (1−*f*)/(1−*e* ^−*n*^). From Lozovaya et al. [62] we know that the inhibition constants (*K_i_*'s) for NR2A- and NR2B-containing NMDARs are 41 and 270 nM. We then ran our simulation at 30°C, the temperature used by Nimchinsky et al. [26], and determined that *P*_ro_ for NR2A- and NR2B-containing NMDARs were 0.70 and 0.20. We then solved for *M*_NR2A_ and *M*_NR2B_, the number of NR2A- and NR2B-containing NMDARs at the synapse, by
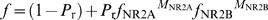
(4)

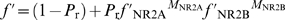
(5)where *f*_NR2X_ = 1−*P*_ro_ and 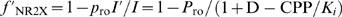, where D-CPP is 240 nM. The equations could also be solved for the case where three receptor species were present, given an additional equation to constrain the number of each species present.

### Receptor Blockade by MK-801

We calculated the average effect of applying 4 msec pulses of 1 mM glutamate and 200 µM MK-801 to NR2A or NR2B-containing receptors using a deterministic, explicit model. We used a doubled version of our NMDAR model, with the second set of states representing having MK-801 bound to the receptor. There was a single, reversible transition between the open and MK-801-bound open states, with forward rate constants of 5.0×10^7^ and 1.39×10^7^/M·sec [44]. To calculate the time-varying probabilities of being in each of the 16 possible states, we constructed a matrix of state-to-state transition probabilities, *P*, for a small time step *dt*. Given 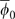, a row vector of the probability distribution over all states at time *t*_0_, we calculated 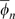 the probability distribution at time *t*_0_+*t*, where *t* = *ndt* by 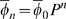. We used a time step of 0.1 µsec for the first 200 msec after simulation and 10 µsec between stimulations.

### LTP Induction

We simulated the effect of applying tetanic stimulation to a synapse containing either NR2A- or NR2B-containing receptors. It was assumed that the presynaptic cell was firing at a rate of 100 Hz for 1 second, but that vesicle release at the synapse was stochastic, with an adapting release probability, modeled using the method of Maass and Zador [84]. For each presynaptic spike, the probability of release, *P*_release_ = 1−*e* ^−*CV*^. The value of *C*, the facilitation parameter, was initially set to *C*_0_. After every presynaptic spike *C* was incremented by *α* and then decayed back towards *C*_0_ exponentially with time constant *τ_C_*. The depletion parameter, *V*, was initially set to *V*_0_. Every time a vesicle was released *V* was decreased by 1, or, if *V*<1, set to 0. *V* then decayed back towards *V*_0_ with time constant *τ_V_*. The parameters *C*_0_, *V*_0_, *τ_C_*, *τ_V_*, and *α* were set to 0.26, 3.5, 20 msec, 50 msec, and 0.25, respectively, based on recordings in hippocampal slices using minimal stimulation [85],[86]. For these simulations, rather than simulate the trajectories of 120,000 glutamate particles, we computed the time-varying average collision rate at each of the 121 possible receptor locations following a single vesicle release by counting collisions and averaging 1000 Monte Carlo runs, and used these averages to randomly determine whether a collision occurred at each time step. Receptor open probability using this technique was indistinguishable from that of the full Monte Carlo simulation (reduced *χ* ^2^ was 1.004 for NR2A- and 0.9617 for NR2B-NMDARs, and *p* values for a paired t tests were 0.9950 and 0.9983, respectively), but the simulations ran ∼2000 times faster.

We modeled internal calcium concentration using the same parameters and model as Nimchinsky et al. [26]. Current was calculated by the Goldman-Hodgkin-Katz equation and concentration showed fast buffering and single exponential decay kinetics. We added a voltage-dependent Mg^2+^ block, fitted to the data of Monyer et al. [83]. Conductance, *G*, was equal to *G*_0_/1+*e* ^−0.08(*V*+20)^, where *G*_0_ is the conductance at 0 mM Mg^2+^ and *V* in voltage in mV. The conductance was half-maximal at −20 mV and increased from 10 to 90 percent over a 54.9 mV range. Calcium concentration was calculated by
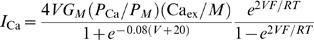
(6)

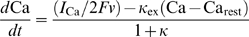
(7)where *I*_Ca_ is calcium current, *G_M_* is the conductance of the channel in the presence of 2 mM Ca^2+^ (46 pS), *P*_Ca_/*P_M_* is the ratio of NMDAR permeability to calcium to permeability to monovalent ions (3.6), Ca_ex_ is external calcium concentration (2 mM), *M* is the concentration of monovalent ions (130 mM), *F* is Faraday's constant, *R* is the gas constant, *T* is temperature, Ca is internal calcium concentration, *v* is spine volume (0.08 fL), *k*_ex_ is the decay rate constant of internal calcium concentration (1.6 msec^−1^), Ca_rest_ is the resting internal calcium concentration (0.1 µM) and *κ* is the buffer capacity (20). *I*_Ca_ was maximal at −14.2 mV. The postsynaptic spine was modeled as a single compartment with uniform concentration throughout. Conductance and calcium permeability were assumed to be the the same for NR2A- and NR2B-NMDARs [10],[82],[97].

The calcium influx through NMDARs leads to the activation of CaMKII which is a significant component of the PSD [113]. The enzyme is activated by the binding of Calcium-Calmodulin (Ca-CaM). When two adjacent subunits bind Ca-CaM, the subunits become autophosphorylated. The kinase then becomes autonomous, that is it retains enzymatic activity even after CaM unbinding. CaMKII is dephosphorylated by Protein phosphatase I (PPI) in a calcium-dependent manner. High levels of calcium can trigger the autophosphorylation of all 12 holoenzymes, which can overcome phosphatase action to dephosphorylate the enzyme. It has been proposed that the dynamics of CaMKII phosphorylation could then function as a bistable “switch”, and that this switch could underly long-term synaptic potentiation [67]. Introduction of active CaMKII in hippocampal neurons mimics LTP [114], and animals with genetic mutations of CaMKII show severe deficits in learning and memory [67]. Based on this evidence, experimental and theoretical efforts have focused on understanding the properties of the CaMKII switch [81], [115]–[118].

We used a bistable model of Ca^2+^/Calmodulin-dependent kinase II (CaMKII) activation [81] as a model of LTP. This was a single-compartment, deterministic model of several interacting chemical processes, driven by the free calcium concentration. For details of the model, and parameter values, see Miller et al. [81]. Essentially, there are two, competing calcium-dependent processes, which phosphorylate and dephosphorylate CaMKII. If the phosphorylation process outcompetes the dephosphorylation process, [CaMKII*] moves towards a high, stable value. We denoted such synapses as potentiated. We ran the simulation for 30 minutes, after which point it was possible to separate the potentiated synapses from those that were not by simply checking whether [CaMKII*] was above a threshold (84 µM).

## References

[1] Bliss TV, Collingridge GL (1993). A synaptic model of memory: long-term potentiation in the hippocampus.. Nature.

[2] Bekkers JM, Stevens CF (1989). NMDA and non-NMDA receptors are co-localized at individual excitatory synapses in cultured rat hippocampus.. Nature.

[3] Kauer JA, Malenka RC, Nicoll RA (1988). NMDA application potentiates synaptic transmission in the hippocampus.. Nature.

[4] Malenka RC, Kauer JA, Perkel DJ, Nicoll RA (1989). The impact of postsynaptic calcium on synaptic transmission—Its role in long-term potentiation.. Trends Neurosci.

[5] Malenka RC, Nicoll RA (1993). NMDA-receptor-dependent synaptic plasticity: multiple forms and mechanisms.. Trends Neurosci.

[6] Malenka RC, Nicoll RA (1999). Long-term potentiation—A decade of progress?. Science.

[7] Daw NW, Stein PS, Fox K (1993). The role of NMDA receptors in information processing.. Annu Rev Neurosci.

[8] Schiller J, Major G, Koester H, Schiller Y (2000). NMDA spikes in basal dendrites of cortical pyramidal neurons.. Nature.

[9] Cull-Candy S, Brickley S, Farrant M (2001). NMDA receptor subunits: diversity, development and disease.. Curr Opin Neurobiol.

[10] Monyer H, Burnashev N, Laurie DJ, Sakmann B, Seeburg PH (1994). Developmental and regional expression in the rat brain and functional properties of four NMDA receptors.. Neuron.

[11] Li JH, Wang YH, Wolfe BB, Krueger KE, Corsi L (1998). Developmental changes in localization of NMDA receptor subunits in primary cultures of cortical neurons.. Eur J Neurosci.

[12] Tovar KR, Westbrook GL (1999). The incorporation of NMDA receptors with a distinct subunit composition at nascent hippocampal synapses in vitro.. J Neurosci.

[13] Townsend M, Yoshii A, Mishina M, Constantine-Paton M (2003). Developmental loss of miniature *N*-methyl-d-aspartate receptor currents in NR2A knockout mice.. Proc Natl Acad Sci U S A.

[14] Thomas CG, Miller AJ, Westbrook GL (2006). Synaptic and extrasynaptic NMDA receptor NR2 subunits in cultured hippocampal neurons.. J Neurophysiol.

[15] Ito I, Kawakami R, Sakimura K, Mishina M, Sugiyama H (2000). Input-specific targeting of NMDA receptor subtypes at mouse hippocampal CA3 pyramidal neuron synapses.. Neuropharmacology.

[16] Kumar SS, Huguenard JR (2003). Pathway-specific differences in subunit composition of synaptic NMDA receptors on pyramidal neurons in neocortex.. J Neurosci.

[17] Sobczyk A, Scheuss V, Svoboda K (2005). NMDA receptor subunit-dependent [Ca^2+^] signaling in individual hippocampal dendritic spines.. J Neurosci.

[18] Cull-Candy SG, Leszkiewicz DN (2004). Role of distinct NMDA receptor subtypes at central synapses.. Sci STKE.

[19] Prybylowski K, Fu Z, Losi G, Hawkins LM, Luo J (2002). Relationship between availability of NMDA receptor subunits and their expression at the synapse.. J Neurosci.

[20] Chen N, Luo T, Raymond LA (1999). Subtype-dependence of NMDA receptor channel open probability.. J Neurosci.

[21] Erreger K, Dravid SM, Banke TG, Wyllie DJA, Traynelis SF (2005). Subunit-specific gating controls rat NR1/NR2A and NR1/NR2B NMDA channel kinetics and synaptic signaling profiles.. J Physiol.

[22] Feng B, Morley R, Jane D, Monaghan D (2005). The effect of competitive antagonist chain length on NMDA receptor subunit selectivity.. Neuropharmacology.

[23] Berberich S, Punnakkal P, Jensen V, Pawlak V, Seeburg PH (2005). Lack of NMDA receptor subtype selectivity for hippocampal long-term potentiation.. J Neurosci.

[24] Frizelle P, Chen P, Wyllie D (2006). Equilibrium constants for (*R*)-[(*S*)-1-(4-bromo-phenyl)-ethylamino]-(2,3-dioxo-1,2,3,4-tetrahydroquinoxalin-5-yl)-methyl]-phosphonic acid (NVPAAM077) acting at recombinant NR1/NR2A and NR1/NR2B *N*-methyl-d-aspartate receptors: implications for studies of synaptic transmission.. Mol Pharmacol.

[25] Oertner TG, Sabatini BL, Nimchinsky EA, Svoboda K (2002). Facilitation at single synapses probed with optical quantal analysis.. Nat Neurosci.

[26] Nimchinsky EA, Yasuda R, Oertner TG, Svoboda K (2004). The number of glutamate receptors opened by synaptic stimulation in single hippocampal spines.. J Neurosci.

[27] Dalby N, Mody I (2003). Activation of NMDA receptors in rat dentate gyrus granule cells by spontaneous and evoked transmitter release.. J Neurophysiol.

[28] Liu L, Wong TP, Pozza MF, Lingenhoehl K, Wang Y (2004). Role of NMDA receptor subtypes in governing the direction of hippocampal synaptic plasticity.. Science.

[29] Lester RA, Jahr CE (1992). NMDA channel behavior depends on agonist affinity.. J Neurosci.

[30] Holmes WR (2000). Models of calmodulin trapping and CaM kinase II activation in a dendritic spine.. J Comput Neurosci.

[31] Franks KM, Bartol TM, Sejnowski TJ (2002). A Monte Carlo model reveals independent signaling at central glutamatergic synapses.. Biophys J.

[32] Kullmann DM, Min MY, Asztely F, Rusakov DA (1999). Extracellular glutamate diffusion determines the occupancy of glutamate receptors at CA1 synapses in the hippocampus.. Philos Trans R Soc Lond B Biol Sci.

[33] Scimemi A, Fine A, Kullmann DM, Rusakov DA (2004). NR2B-containing receptors mediate cross talk among hippocampal synapses.. J Neurosci.

[34] Popescu G, Auerbach A (2003). Modal gating of NMDA receptors and the shape of their synaptic response.. Nat Neurosci.

[35] Popescu G, Robert A, Howe JR, Auerbach A (2004). Reaction mechanism determines NMDA receptor response to repetitive stimulation.. Nature.

[36] Raghavachari S, Lisman JE (2004). Properties of quantal transmission at CA1 synapses.. J Neurophysiol.

[37] Bartol TM, Land BR, Salpeter EE, Salpeter MM (1991). Monte Carlo simulation of miniature endplate current generation in the vertebrate neuromuscular junction.. Biophys J.

[38] Wahl LM, Pouzat C, Stratford KJ (1996). Monte Carlo simulation of fast excitatory synaptic transmission at a hippocampal synapse.. J Neurophysiol.

[39] Rosenmund C, Stern-Bach Y, Stevens CF (1998). The tetrameric structure of a glutamate receptor channel.. Science.

[40] Armstrong N, Gouaux E (2000). Mechanisms for activation and antagonism of an AMPA-sensitive glutamate receptor: crystal structures of the GluR2 ligand binding core.. Neuron.

[41] Sun Y, Olson R, Horning M, Armstrong N, Mayer M (2002). Mechanism of glutamate receptor desensitization.. Nature.

[42] Robert A, Howe JR (2003). How AMPA receptor desensitization depends on receptor occupancy.. J Neurosci.

[43] Harris KM, Stevens JK (1989). Dendritic spines of CA 1 pyramidal cells in the rat hippocampus: serial electron microscopy with reference to their biophysical characteristics.. J Neurosci.

[44] Dravid SM, Erreger K, Yuan H, Nicholson K, Le P (2007). Subunit-specific mechanisms and proton sensitivity of NMDA receptor channel block.. J Physiol.

[45] Tong G, Jahr CE (1994). Multivesicular release from excitatory synapses of cultured hippocampal neurons.. Neuron.

[46] Christie JM, Jahr CE (2006). Multivesicular release at Schaffer collateral-CA1 hippocampal synapses.. J Neurosci.

[47] Chavis P, Westbrook G (2001). Integrins mediate functional pre- and postsynaptic maturation at a hippocampal synapse.. Nature.

[48] Hsia A, Malenka R, Nicoll R (1998). Development of excitatory circuitry in the hippocampus.. J Neurophysiol.

[49] Mainen ZF, Malinow R, Svoboda K (1999). Synaptic calcium transients in single spines indicate that NMDA receptors are not saturated.. Nature.

[50] Tovar KR, Westbrook GL (2002). Mobile NMDA receptors at hippocampal synapses.. Neuron.

[51] Ivanov A, Pellegrino C, Rama S, Dumalska I, Salyha Y (2006). Opposing role of synaptic and extrasynaptic NMDA receptors in regulation of the ERK activity in cultured rat hippocampal neurons.. J Physiol.

[52] Arnth-Jensen N, Jabaudon D, Scanziani M (2002). Cooperation between independent hippocampal synapses is controlled by glutamate uptake.. Nat Neurosci.

[53] Harris A, Pettit D (2008). Recruiting extrasynaptic NMDA receptors augments synaptic signaling.. J Neurophysiol.

[54] Barbour B (2001). An evaluation of synapse independence.. J Neurosci.

[55] Rusakov D, Kullmann D (1998). Extrasynaptic glutamate diffusion in the hippocampus: ultrastructural constraints, uptake, and receptor activation.. J Neurosci.

[56] Racca C, Stephenson FA, Streit P, Roberts JD, Somogyi P (2000). NMDA receptor content of synapses in stratum radiatum of the hippocampal CA1 area.. J Neurosci.

[57] Wu Y, Kawakami R, Shinohara Y, Fukaya M, Sakimura K (2005). Target-cell-specific left-right asymmetry of NMDA receptor content in schaffer collateral synapses in epsilon1/NR2A knock-out mice.. J Neurosci.

[58] Skeberdis VA, Chevaleyre V, Lau CG, Goldberg JH, Pettit DL (2006). Protein kinase A regulates calcium permeability of NMDA receptors.. Nat Neurosci.

[59] Raastad M (1995). Extracellular activation of unitary excitatory synapses between hippocampal CA3 and CA1 pyramidal cells.. Eur J Neurosci.

[60] Matsuzaki M, Ellis-Davies GC, Nemoto T, Miyashita Y, Iino M (2001). Dendritic spine geometry is critical for AMPA receptor expression in hippocampal CA1 pyramidal neurons.. Nat Neurosci.

[61] Matsuzaki M, Honkura N, Ellis-Davies GCR, Kasai H (2004). Structural basis of long-term potentiation in single dendritic spines.. Nature.

[62] Lozovaya NA, Grebenyuk SE, Tsintsadze TS, Feng B, Monaghan DT (2004). Extrasynaptic NR2B and NR2D subunits of NMDA receptors shape ‘superslow’ afterburst EPSC in rat hippocampus.. J Physiol.

[63] Sheng M, Cummings J, Roldan LA, Jan YN, Jan LY (1994). Changing subunit composition of heteromeric NMDA receptors during development of rat cortex.. Nature.

[64] Al-Hallaq RA, Conrads TP, Veenstra TD, Wenthold RJ (2007). NMDA di-heteromeric receptor populations and associated proteins in rat hippocampus.. J Neurosci.

[65] Kew J, Richards J, Mutel V, Kemp J (1998). Developmental changes in NMDA receptor glycine affinity and ifenprodil sensitivity reveal three distinct populations of NMDA receptors in individual rat cortical neurons.. J Neurosci.

[66] Hatton CJ, Paoletti P (2005). Modulation of triheteromeric NMDA receptors by N-terminal domain ligands.. Neuron.

[67] Lisman J, Schulman H, Cline H (2002). The molecular basis of CaMKII function in synaptic and behavioural memory.. Nat Rev Neurosci.

[68] Colbran RJ (2004). Protein phosphatases and calcium/calmodulin-dependent protein kinase II-dependent synaptic plasticity.. J Neurosci.

[69] Shi S, Hayashi Y, Petralia R, Zaman S, Wenthold R (1999). Rapid spine delivery and redistribution of AMPA receptors after synaptic NMDA receptor activation.. Science.

[70] Hayashi Y, Shi S, Esteban J, Piccini A, Poncer J (2000). Driving AMPA receptors into synapses by LTP and CaMKII: requirement for GluR1 and PDZ domain interaction.. Science.

[71] Lee HK, Takamiya K, Han JS, Man H, Kim CH (2003). Phosphorylation of the AMPA receptor GluR1 subunit is required for synaptic plasticity and retention of spatial memory.. Cell.

[72] Sutton MA, Ito HT, Cressy P, Kempf C, Woo JC (2006). Miniature neurotransmission stabilizes synaptic function via tonic suppression of local dendritic protein synthesis.. Cell.

[73] Hardingham G, Arnold F, Bading H (2001). A calcium microdomain near NMDA receptors: on switch for ERK-dependent synapse-to-nucleus communication.. Nat Neurosci.

[74] Krapivinsky G, Krapivinsky L, Manasian Y, Ivanov A, Tyzio R (2003). The NMDA receptor is coupled to the ERK pathway by a direct interaction between NR2B and RasGRF1.. Neuron.

[75] Kim MJ, Dunah AW, Wang YT, Sheng M (2005). Differential roles of NR2A- and NR2B-containing NMDA receptors in Ras-ERK signaling and AMPA receptor trafficking.. Neuron.

[76] Weitlauf C, Honse Y, Auberson YP, Mishina M, Lovinger DM (2005). Activation of NR2A-containing NMDA receptors is not obligatory for NMDA receptor-dependent long-term potentiation.. J Neurosci.

[77] Tang YP, Shimizu E, Dube GR, Rampon C, Kerchner GA (1999). Genetic enhancement of learning and memory in mice.. Nature.

[78] Barria A, Malinow R (2005). NMDA receptor subunit composition controls synaptic plasticity by regulating binding to CaMKII.. Neuron.

[79] Toyoda H, Zhao M, Zhuo M (2005). Roles of NMDA receptor NR2A and NR2B subtypes for long-term depression in the anterior cingulate cortex.. Eur J Neurosci.

[80] Yang SN, Tang YG, Zucker RS (1999). Selective induction of LTP and LTD by postsynaptic [Ca^2+^]i elevation.. J Neurophysiol.

[81] Miller P, Zhabotinsky AM, Lisman JE, Wang XJ (2005). The stability of a stochastic CaMKII switch: dependence on the number of enzyme molecules and protein turnover.. PLoS Biol.

[82] Stern P, Béhé P, Schoepfer R, Colquhoun D (1992). Single-channel conductances of NMDA receptors expressed from cloned cDNA: comparison with native receptors.. Proc R Soc Lond B.

[83] Monyer H, Sprengel R, Schoepfer R, Herb A, Higuchi M (1992). Heteromeric NMDA receptors: molecular and functional distinction of subtypes.. Science.

[84] Maass W, Zador AM (1999). Dynamic stochastic synapses as computational units.. Neural Comput.

[85] Stevens CF, Wang Y (1995). Facilitation and depression at single central synapses.. Neuron.

[86] Dobrunz LE, Stevens CF (1997). Heterogeneity of release probability, facilitation, and depletion at central synapses.. Neuron.

[87] Dittman JS, Kreitzer AC, Regehr WG (2000). Interplay between facilitation, depression, and residual calcium at three presynaptic terminals.. J Neurosci.

[88] Markram H, Lübke J, Frotscher M, Sakmann B (1997). Regulation of synaptic efficacy by coincidence of postsynaptic APs and EPSPs.. Science.

[89] Kadotani H, Hirano T, Masugi M, Nakamura K, Nakao K (1996). Motor discoordination results from combined gene disruption of the NMDA receptor NR2A and NR2C subunits, but not from single disruption of the NR2A or NR2C subunit.. J Neurosci.

[90] Noguchi J, Matsuzaki M, Ellis-Davies GCR, Kasai H (2005). Spine-neck geometry determines NMDA receptor-dependent Ca^2+^ signaling in dendrites.. Neuron.

[91] Bekkers J, Richerson G, Stevens C (1990). Origin of variability in quantal size in cultured hippocampal neurons and hippocampal slices.. Proc Natl Acad Sci U S A.

[92] Scanziani M, Salin PA, Vogt KE, Malenka RC, Nicoll RA (1997). Use-dependent increases in glutamate concentration activate presynaptic metabotropic glutamate receptors.. Nature.

[93] Savtchenko LP, Rusakov DA (2005). Extracellular diffusivity determines contribution of high versus low-affinity receptors to neural signaling.. Neuroimage.

[94] Jourdain P, Bergersen LH, Bhaukaurally K, Bezzi P, Santello M (2007). Glutamate exocytosis from astrocytes controls synaptic strength.. Nat Neurosci.

[95] Bartlett TE, Bannister NJ, Collett VJ, Dargan SL, Massey PV (2007). Differential roles of NR2A and NR2B-containing NMDA receptors in LTP and LTD in the CA1 region of two-week old rat hippocampus.. Neuropharmacology.

[96] Berberich S, Jensen V, Hvalby Ø, Seeburg PH, Köhr G (2007). The role of NMDAR subtypes and charge transfer during hippocampal LTP induction.. Neuropharmacology.

[97] Schneggenburger R (1996). Simultaneous measurement of Ca^2+^ influx and reversal potentials in recombinant *N*-methyl-d-aspartate receptor channels.. Biophys J.

[98] Yasuda H, Barth A, Stellwagen D, Malenka R (2003). A developmental switch in the signaling cascades for LTP induction.. Nat Neurosci.

[99] Harris KM, Teyler TJ (1984). Developmental onset of long-term potentiation in area CA1 of the rat hippocampus.. J Physiol.

[100] Song S, Miller KD, Abbott LF (2000). Competitive Hebbian learning through spike-timingdependent synaptic plasticity.. Nat Neurosci.

[101] Song S, Abbott L (2001). Cortical development and remapping through spike timingdependent plasticity.. Neuron.

[102] Sobczyk A, Svoboda K (2007). Activity-dependent plasticity of the NMDA-receptor fractional Ca^2+^ current.. Neuron.

[103] Philpot BD, Cho KKA, Bear MF (2007). Obligatory role of NR2A for metaplasticity in visual cortex.. Neuron.

[104] Bellone C, Nicoll RA (2007). Rapid bidirectional switching of synaptic NMDA receptors.. Neuron.

[105] Nielsen TA, DiGregorio DA, Silver RA (2004). Modulation of glutamate mobility reveals the mechanism underlying slow-rising AMPAR EPSCs and the diffusion coefficient in the synaptic cleft.. Neuron.

[106] Longsworth L (1953). Diffusion measurements, at 25°, of aqueous solutions of amino acids, peptides and sugars.. J Am Chem Soc.

[107] Magee JC, Cook EP (2000). Somatic EPSP amplitude is independent of synapse location in hippocampal pyramidal neurons.. Nat Neurosci.

[108] Jonas P, Major G, Sakmann B (1993). Quantal components of unitary EPSCs at the mossy fibre synapse on CA3 pyramidal cells of rat hippocampus.. J Physiol.

[109] Andrasfalvy B, Magee J (2001). Distance-dependent increase in AMPA receptor number in the dendrites of adult hippocampal CA1 pyramidal neurons.. J Neurosci.

[110] Hestrin S, Sah P, Nicoll R (1990). Mechanisms generating the time course of dual component excitatory synaptic currents recorded in hippocampal slices.. Neuron.

[111] Lehre K, Danbolt N (1998). The number of glutamate transporter subtype molecules at glutamatergic synapses: chemical and stereological quantification in young adult rat brain.. J Neurosci.

[112] Grewer C, Watzke N, Wiessner M, Rauen T (2000). Glutamate translocation of the neuronal glutamate transporter EAAC1 occurs within milliseconds.. Proc Natl Acad Sci U S A.

[113] Chen X, Vinade L, Leapman RD, Petersen JD, Nakagawa T (2005). Mass of the postsynaptic density and enumeration of three key molecules.. Proc Natl Acad Sci U S A.

[114] Lledo PM, Hjelmstad GO, Mukherji S, Soderling TR, Malenka RC (1995). Calcium/calmodulin-dependent kinase II and long-term potentiation enhance synaptic transmission by the same mechanism.. Proc Natl Acad Sci U S A.

[115] Kubota Y, Bower JM (2001). Transient versus asymptotic dynamics of CaM kinase II: possible roles of phosphatase.. J Comput Neurosci.

[116] Lisman JE, Zhabotinsky AM (2001). A model of synaptic memory: a CaMKII/PP1 switch that potentiates transmission by organizing an AMPA receptor anchoring assembly.. Neuron.

[117] D'Alcantara P, Schiffmann SN, Swillens S (2003). Bidirectional synaptic plasticity as a consequence of interdependent Ca^2+^-controlled phosphorylation and dephosphorylation pathways.. Eur J Neurosci.

[118] Hayer A, Bhalla US (2005). Molecular switches at the synapse emerge from receptor and kinase traffic.. PLoS Comput Biol.

[119] Burger P, Mehl E, Cameron P, Maycox P, Baumert M (1989). Synaptic vesicles immunoisolated from rat cerebral cortex contain high levels of glutamate.. Neuron.

